# Light‐intensity and moderate‐to‐vigorous intensity physical activity among older adult breast cancer survivors with obesity: A narrative review

**DOI:** 10.1002/cam4.4841

**Published:** 2022-05-26

**Authors:** Brett R. Gordon, Maxime Caru, Cindy K. Blair, Shirley M. Bluethmann, David E. Conroy, Shawna E. Doerksen, Jonathon G. Hakun, Kathleen M. Sturgeon, Melanie Potiaumpai, Christopher N. Sciamanna, Kathryn H. Schmitz

**Affiliations:** ^1^ Penn State College of Medicine Hershey Pennsylvania USA; ^2^ Department of Internal Medicine University of New Mexico Albuquerque New Mexico USA; ^3^ University of New Mexico Comprehensive Cancer Center Albuquerque New Mexico USA; ^4^ The Pennsylvana State University, University Park Pennsylvania USA; ^5^ Northwestern University Chicago Illinois USA

**Keywords:** aged, health promotion, breast neoplasms, exercise, obesity

## Abstract

**Background:**

With an aging population, rising incidence of breast cancer, improved survival rates, and obesity epidemic, there will be a growing population of older adult breast cancer survivors with obesity. This complex population, often with multimorbidity, is at risk for several poor health outcomes, including recurrence, cardiovascular disease, dementia, and diabetes, and a number of deleterious symptoms, including a worsened inflammatory profile, breast cancer‐ related lymphedema, mobility disability, cognitive impairment, anxiety, and depressive symptoms. A wealth of meta‐analytic and randomized controlled trial evidence show that adherence to World Health Organization and 2018 United States Physical Activity guidelines‐based levels of moderate‐to‐vigorous physical activity (MVPA) reduces risk of all‐cause mortality, and improves symptoms. However, few survivors engage in recommended levels of MVPA, and symptoms related to their multimorbidity may preclude engaging in sufficient levels of MVPA. Additional research of MVPA in this population is warranted; however, understudied light‐intensity physical activity (LIPA) may be a more pragmatic target than MVPA among this complex population facing extensive challenges meeting MVPA recommendations. Large benefits are likely to occur from increasing these survivors' total activity, and LIPA prescriptions may be a more pragmatic approach than MVPA to aid this transition.

**Methods:**

We present a broad, narrative review of the evidence for MVPA and LIPA in this population on an array of health outcomes across the translational science spectrum (clinical, implementation, and public health), and identify a number of directions for future research focused on understanding the potential diverse health effects of LIPA.

**Conclusion:**

Additional LIPA research is warranted, as LIPA prescriptions may be a pragmatic strategy to effectively promote physical activity to this complex population.

## 1 INTRODUCTION

More than 50% of breast cancer survivors are over the age of 60 years.^1^ Many breast cancer survivors either are currently overweight or have obesity. Approximately one fifth of breast cancer survivors diagnosed ages 65–92 years are obese.^2^, ^3^ Older adult breast cancer survivors have decreased health‐related quality of life and psychological well‐being compared to younger survivors,^4^ and breast cancer survivors with obesity pre‐ and post‐diagnosis have worse disease‐free and overall survival, experience more complications related to treatment, and are at increased risk for local recurrence compared to nonobese breast cancer survivors.^5^

The health benefits of moderate‐to‐vigorous intensity physical activity (MVPA) for cancer survivors, older adults, and people with obesity are compelling, yet few members of these groups engage in recommended levels of MVPA.^6^ People belonging to more than one of these groups, such as older adult breast cancer survivors with obesity, may have particular needs for alternative interventions to increase health‐enhancing physical activity. Light‐intensity physical activity (LIPA) may be a more pragmatic prescription than MVPA among this complex population with multimorbidity facing extensive challenges meeting existing MVPA recommendations, including barriers related to their age, obesity, and cancer. Definitions for terminology used are described in Table 1.

**TABLE 1:** Definitions of terminology

Terminology	Definition
Moderate‐to‐vigorous intensity physical activity	Regarding absolute intensity, physical activity conducted at 3.0 to 5.9 METs. Regarding relative intensity, activity at a level of effort of 5 or greater on a scale of 0 to 10, where 0 is the level of effort of sitting, and 10 is the maximal effort.^8^
Light‐intensity physical activity	Regarding absolute intensity, non‐sedentary behaviors of less than 3.0 METs. Regarding relative intensity, a level of effort of <5 on a scale of 0–10, where 0 is the level of effort of sitting, and 10 is the maximal effort.^8^
Obese	Body mass index ≥30^64^
Older adult	Varying ages have been used as the cutoff point for older adults. The United States Physical activity guidelines uses 65 years and older to refer to older adults.^8^

Abbreviation: MET, metabolic equivalent of a task.

Physical activity guidelines dating as far back as 1978 called for engaging in physical activity at moderate‐intensity or greater.^7^ Both the 2008 and 2018 US Physical Activity Guidelines recommended adults get at least 150 min of moderate‐intensity aerobic physical activity, or 75 min of vigorous‐intensity physical activity, or an equivalent combination, per week.^8^, ^9^ The guidelines include supplemental messaging such as “avoid inactivity,” and “some physical activity is better than none,” but no numerical target for LIPA. Authors of the guidelines did not establish numerical targets for LIPA for many reasons, including finding insufficient evidence linking LIPA volume with decreased mortality risk.^8^

Accelerometry‐based evidence indicated only 11% of breast cancer survivors met MVPA guidelines, and cancer survivors (all sites) with obesity engaged in 47% less MVPA than normal weight survivors.^10^, ^11^ Both of these datasets comprised populations of predominately older adults. The low levels of MVPA among older adult breast cancer survivors with obesity raise questions whether guidelines emphasizing MVPA exclusively could be expanded. Insufficiently active survivors are likely to experience large health benefits beyond decreased mortality risk moving from being inactive to active, and LIPA may be a more pragmatic approach to this transition. In this manuscript, we highlight the importance of this population, the available evidence of MVPA and LIPA to improve health in this population, and identify targeted directions for future research.

### 1.1 A growing need to support inactive, older adult breast cancer survivors with obesity

Breast cancer is the most frequently diagnosed cancer; about 290,560 new cases (both sexes) are diagnosed in the US every year.^12^ Due to earlier detection, improved treatment, and an aging population, incidence is rising and expected to continue.^13^ The most important risk factor for breast cancer is age; breast cancer incidence and death rates increase with age, and the median age at time of diagnosis is 62 years.^13^ In 2016, 62% of all cancer survivors were aged 65 years or older, and that is expected to rise to 73% by 2040.^14^ The persistent obesity epidemic will also contribute to a growing population of inactive, older adult breast cancer survivors with obesity. In a recent large cohort study, approximately 20% of 4069 breast cancer survivors diagnosed between the ages of 65–92 years were obese.^3^ Among breast cancer survivors, both excess weight pre‐diagnosis, and weight gain following diagnoses are associated with recurrence and death.^15^ Beyond the growing number of older adults with obesity who will be diagnosed with breast cancer, women of normal weight may become obese following diagnosis; in a study of 277 breast cancer survivors, half (*n* = 140) gained at least 10 pounds, with 17% (*n* = 47) gaining more than 44 pounds after diagnosis. Among this sample, 40 women (14.8%) went from an overweight BMI classification to obese, and five women (1.9%) went from a healthy BMI classification to obese.^2^

Due to their cancer, obesity, age, and inactivity, these breast cancer survivors are at increased risk for several poor health outcomes, including recurrence, cardiovascular disease, dementia, and diabetes.^14^, ^16^, ^17^, ^18^ They are also at risk for associated deleterious symptoms (worsened inflammatory profile, mobility disability, cognitive impairment, anxiety, and depressive symptoms),^19^, ^20^, ^21^, ^22^ furthering their risk for these poor health outcomes. Clearly, there will be a growing population of inactive, older adult breast cancer survivors with obesity requiring accessible and appealing strategies to improve health, including physical activity.

### 1.2 The MVPA and LIPA levels of older adult breast cancer survivors with obesity

MVPA levels are low in the general population, and being a woman, an older adult, a breast cancer survivor, and having obesity are all individually associated with lower levels of MVPA. In the most recent NHANES cohort (2007–2017) assessing self‐reported physical activity, older adults were the least adherent to MVPA guidelines, women were less adherent than men, and individuals with obesity were less adherent than overweight or normal weight individuals.^6^ Accelerometer‐based evidence from NHANES indicated adults accumulate approximately 16 unbouted minutes of MVPA per day,^23^ older women (60–69, 70–79, ≥80 years) engaged in less MVPA,^24^ and only 9% of breast cancer survivors met MVPA guidelines. Further, cancer survivors (all sites) with obesity engaged in 47% less MVPA than survivors with normal weight.^10^ Among 1535 cancer survivors (mean age = 65.1, standard error = 0.4 years) self‐reporting physical activity levels, 57% engaged in no leisure‐time physical activity, and an additional 16% engaged in <150 min of leisure‐time physical activity per week.^25^

Waking sedentary behavior and LIPA are linked in a zero‐sum time use relationship with overall physical activity, although distinctions can be made in strategies to increase LIPA or reduce sedentary behavior. Inherently, reducing sedentary behavior involves increasing LIPA.^26^ In NHANES, accelerometry‐based evidence showed adults accumulated 322 min of daily LIPA, and 533 min of daily sedentary time, in comparison to 16 min of MVPA.^23^ Other NHANES accelerometry evidence showed older women engage in more sedentary behavior, longer bouts of sedentary behavior, and less LIPA compared to younger women.^24^ Further, cancer survivors (all sites) with obesity engaged in comparatively less LIPA minutes per day (281.9 ± 17.2) compared to survivors with overweight (301.8 ± 11.6) or normal weight (302 ± 17.4).^10^ A recent NHANES cohort study examining self‐reported sitting‐time and leisure‐time physical activity among 1535 cancer survivors showed that prolonged sitting with lack of physical activity was associated with the highest risks of death from all causes and cancer.^25^ As MVPA levels in this population are low, moving the needle on physical activity via positive changes in both LIPA and sedentary behavior in tandem may be a more pragmatic approach to improve health in this population.

### 1.3 Potential barriers to MVPA among older adult breast cancer survivors with obesity

There are a number of actual and perceived barriers to MVPA common among older adults, individuals with obesity, breast cancer survivors, and combinations of those populations. Cross‐sectional evidence among 145 self‐reportedly inactive older women cited poor health (59%), lack of company (48%), lack of interest (27%), and lack of opportunities (30%) as primary barriers to physical activity.^27^ Beyond age, there are a number of actual and perceived barriers to MVPA among individuals with obesity. A recent rapid evidence assessment identified 17 studies focused on unique barriers to MVPA faced by individuals with obesity. Barriers included physical barriers such as excess weight and poor fitness, psychological barriers such as weight perception, and external barriers such as lack of time and knowledge.^28^

Barriers also could arise from these individuals' breast cancer, including mobility disability, and pain‐related disability.^29^ Breast cancer survivors specifically deal with unique ambulatory disabilities, including fear of/presence of lymphedema, aromatase inhibitor‐induced arthralgia, and functional limitations involving the use of the arm, contributing to limitations in activities of daily life.^30^, ^31^ Cross‐sectional evidence indicated cancer survivors (all sites) who perceived themselves as having disabilities were 50% less likely to participate in physical activity than those who did not.^32^ Multimorbidity may also further reduce MVPA.^32^ Comorbidities, such as diabetes, congestive heart failure, and chronic obstructive pulmonary disease, are common (17%) in survivors, and older cancer survivors (all sites) with comorbidity are less than half as likely to meet MVPA guidelines.^14^ Other cancer‐specific actual and perceived barriers include a fear of “overdoing” physical activity.^29^ Fear of “overdoing” physical activity, and perceptions of disability, may be re‐enforced through society or healthcare providers, as survivors historically have been told to avoid or limit certain physical activity and resistance exercises because of the risk of developing/worsening lymphedema. This advice could be interpreted incorrectly as advice to avoid any physical activity.^33^ Given the low percentage of older adult breast cancer survivors with obesity meeting MVPA guidelines, and the diverse real and perceived barriers to MVPA, alternative strategies are needed to evaluate and address the perceived and actual barriers to physical activity among older adult breast cancer survivors with obesity. LIPA may be an alternative strategy for engaging in physical activity if MVPA is too strenuous or undesirable for some survivors.

We present a broad narrative review of the evidence for MVPA and LIPA in this population on an array of health outcomes, highlighting the impact along the relevant translational science spectrum (*Clinical*, *Implementation*, and *Public Health*).^34^ Findings and a proposed agenda for MVPA and LIPA research in this population are briefly summarized in Table 2.

**TABLE 2:** State of the current evidence and need for future research

	MVPA	LIPA	Future directions
Clinical	Clear evidence of benefits of MVPA across diverse range of health outcomes among breast cancer survivors generally, but limited trials focused on older adult breast cancer survivors with obesity. Although findings of improved health outcomes are potentially generalizable to this population, important outcomes such as acceptability, adherence, and compliance may not be as generalizable.	Few deliberate LIPA trials at all, and no trials focused on older adult breast cancer survivors with obesity.	RCTs of LIPA and MVPA on an array of health outcomes among older adult breast cancer survivors with obesity, particularly trials reporting outcomes such as acceptability, adherence, and compliance to physical activity interventions. LIPA may not be as efficacious as MVPA to improve various health outcomes, but may be a more tolerable, enjoyable, and accessible strategy to increase physical activity while also minimizing sedentary behavior to improve health.
Implementation	Clear evidence of effectiveness of guidelines‐based MVPA programs among breast cancer survivors generally, but limited evidence among older adult breast cancer survivors with obesity specifically.	No large efficacy trials or effectiveness trials focused on increasing LIPA have been conducted.	Appropriately powered effectiveness trials examining a range of implementation outcomes.
Public Health	Clear public health guidelines from diverse organizations for at least 150 min of MVPA per week (or 75 min of vigorous physical activity/equivalent combination). Established benefits for diverse health outcomes, yet general population levels of MVPA are low, and undoubtedly low among older adult breast cancer survivors with obesity.	No clear guidelines for LIPA beyond “avoid inactivity.” No numerical targets for LIPA. Epidemiological associations of benefits of increase LIPA across diverse health outcomes, but little trial‐based evidence underpinning recommendations.	Numerical targets for LIPA for diverse health effects including and/or beyond recurrence and mortality alone.

Abbreviations: LIPA, Light‐ intensity physical activity; MVPA, Moderate‐to‐vigorous intensity physical activity.

### 1.4 The known value of MVPA for older adult breast cancer survivors with obesity

#### 1.4.1 Clinical

In a recent Cochrane review of 63 RCTs examining the effects of physical activity among women with breast cancer after adjuvant therapy (including diverse physical activity interventions of all intensities), MVPA significantly improved health‐related quality of life (standardized mean difference [SMD] = 0.78), emotion function (SMD = 0.31), perceived physical function (SMD = 0.60), anxiety (SMD = ‐0.37), and cardiorespiratory fitness (SMD = 0.83).^35^ There were no sub‐group analyses by body‐composition at baseline. No RCTs examined the effects of physical activity after cancer treatment on risk of recurrence; however, a recent review of follow‐ups to two large RCTs of MVPA among breast cancer survivors support the hypothesis post‐diagnosis exercise during and beyond breast cancer treatment can improve breast cancer outcomes, potentially including recurrence.^36^ Further, several cohort studies have shown post‐diagnosis physical activity is inversely associated with breast cancer‐specific mortality, and all‐cause mortality.^3^, ^37^

The Cochrane review also pointed toward the positive effects among older survivors, and survivors with obesity. Participants' mean age was 58 years, and the mean body mass index (BMI) was 28 kg/m^2^ (>30 kg/m^2^ is obese). A recent, small RCT among breast cancer survivors with obesity indicated a multimodal lifestyle intervention including a MVPA guidelines‐based exercise component improved BMI, total physical activity, and VO_2max_.^38^ Another recent small RCT in older adult breast cancer survivors whose BMI ranged from overweight to obese indicated a MVPA aerobic plus resistance training program improved patient‐reported outcomes.^39^ MVPA is clearly, and robustly, an effective and health‐enhancing intervention for older adult breast cancer survivors with obesity.

#### 1.4.2 Implementation

Implementation research focuses on working within real‐world conditions, rather than highly supervised RCTs, including assessment of acceptability, fidelity, cost, sustainability, coverage, etc.^40^ Despite the clear benefits of MVPA, adherence to MVPA interventions can be low, and RCTs among breast cancer survivors commonly experience barriers to recruiting and retaining participants.^41^ A recent systematic review of the effects of diverse physical activity interventions on implementation outcomes among breast cancer survivors examined 17 interventions, and a disparity in reporting of interventions limited the ability to determine which facets of physical activity, including intensity, facilitated the best participation, and improvement across a range of outcomes.^42^ In a recent systematic review of the determinants of exercise adherence and maintenance among cancer survivors (all sites), a lack of studies utilizing varying ranges of intensities limited the ability to examine if adherence was moderated by exercise intensity.^43^ Beyond solely adherence, a recent review of implementation trials among physical activity interventions for cancer survivors demonstrated the heterogeneity of implementation outcome reporting in effectiveness trials, including acceptability, adoption, appropriateness, cost, feasibility, fidelity, penetration, and sustainability.^44^

#### 1.4.3 Public health

The authors of the Exercise Guidelines for Cancer Survivors: A Consensus Statement from an International Multidisciplinary Roundtable examined the evidence from more than 2500 published randomized controlled trials (RCTs) of physical activity for a number of physical and mental health outcomes. The available evidence clearly indicates MVPA is safe and effective for cancer survivors, and improves common cancer‐related health outcomes, including anxiety, depressive symptoms, fatigue, physical functioning, and health‐related quality of life.^45^ A key limitation highlighted in the Consensus Statement from the International Multidisciplinary Roundtable were the lack of trials directly comparing two or more levels of exercise training, such as MVPA versus LIPA.

There are a number of organizational guidelines promoting MVPA to breast cancer survivors.^8^, ^45^, ^46^, ^47^ In the Consensus Statement from International Multidisciplinary Roundtable, specific FITT (frequency, intensity, time, and type) prescriptions are available for MVPA for individual health outcomes. Inherently, as a majority of the evidence consists of guidelines‐based, homogeneous MVPA protocols, a majority of the FITT prescriptions are for five times per week MVPA (30 min of moderate‐intensity aerobic exercise three times per week, and resistance exercise twice weekly).^45^ The Consensus Statement also comment that weight gain can be a common side effect of breast cancer treatment,^48^ and exercise professionals should be aware of safety considerations related to orthopedic limitations, and cardiovascular disease risk, when prescribing exercise.^45^ Further, it clarifies subgroups of cancer survivors may not be able to tolerate these MVPA prescriptions, and below‐guidelines volumes of physical activity (min/week) should be given to these individuals. Importantly, the lack of trials examining LIPA limits our understanding of the diverse health effects of varying intensities of physical activity.^45^ Beyond public health guidelines, there are a number of organizations and community‐based exercise programs focused on providing support to people affected by cancer through exercise and physical activity. These include organizations that promote physical activity among cancer survivors,^49^, ^50^ and make exercise prescription standard practice in oncology.^51^

### 1.5 The untapped value of LIPA for older adult breast cancer survivors with obesity

#### 1.5.1 Clinical

A clear limitation when examining this literature is the disparate ways intensity is measured and reported. Intensity can be measured in absolute intensity (defined in terms of metabolic equivalent of a task: MET), or relative intensity (percentage of a person's maximal heart rate, heart rate reserve, aerobic capacity reserve, or rate of perceived effort).^8^ Regarding absolute intensity, LIPA is defined as waking non‐sedentary behaviors of less than three METs.^8^ Regarding relative intensity, LIPA is <40% of aerobic capacity reserve, or simply <5 on a scale of 0 (sitting) to 10 (maximal effort). Importantly, the 2018 US Physical Activity Guidelines recommends using relative intensity for older adults with low levels of fitness initially, because “activities in the range of 3.0 to 5.9 METs will be relatively vigorous or physiologically impossible.”^8^ When designing interventions targeting increased LIPA in real settings, utilizing “<5 on the 0‐10 relative intensity scale” may be particularly useful to easily communicate the intended intensity of activities.

Importantly, few trials have been designed to increase LIPA among older adults, individuals with obesity, breast cancer survivors, or combinations of those categories. In a recent Cochrane review of 63 RCTs (including all intensities) among women with breast cancer after adjuvant therapy, researchers utilized a number of varying methods to set and describe the exercise intervention intensity, including percentage of maximum heart rate, target heart rate using the Karvonen formula, the intensity that elicits a specific blood lactate concentration, direct measurement of VO_2_ max, rate of perceived exertion, and subjective intensity.^35^ These disparate descriptions of intensities limit the ability to examine the unique effect of LIPA exclusively. Interestingly, in that review, what authors described as “light‐to‐moderate intensity” interventions improved a range of physical and mental health outcomes to a greater magnitude in comparison to control conditions than the magnitude of change of the “moderate‐to‐high” intensity interventions in comparison to control conditions.^35^ Larger improvements were found among the “light‐to‐moderate intensity” for overall health‐related quality of life, physical function, cognitive function, sexual function, anxiety, depression, fatigue, pain, cardiorespiratory fitness, and both self‐reported and objective physical activity.^35^ The mean age of the included participants in the review was 58 years; however, there was some indication of positive effect among older adults in comparison to younger participants. There was a significant effect of physical activity on health‐related quality of life among “postmenopausal‐only” studies in comparison to not postmenopausal‐only.^35^ There is initial evidence LIPA interventions can actually increase LIPA, and have positive health effects in older adults and cancer survivors, including improving physical performance.^52^, ^53^ Similar to other physical activity interventions, depending on inclusion criteria, such as baseline activity levels, LIPA RCTs may struggle to actually increase LIPA.^53^

#### 1.5.2 Implementation

There have only been small, pilot RCTs focused on LIPA which showed that these small interventions are feasible, acceptable, and did increase LIPA,^52^, ^53^ but no large efficacy trials or effectiveness trials. As such, there is little evidence of its effectiveness. The few, small, disparate RCTs limit drawing evidence‐based conclusions of LIPA's acceptability, and varying implementation outcomes.

#### 1.5.3 Public health

The US Physical Activity guidelines promoted concise, easily communicable targets for weekly minutes of moderate‐intensity physical activity (150 min), and vigorous intensity physical activity (75 min). These guidelines are reflected in various cancer‐related organizations as well.^54^, ^55^ Guidelines include supplemental messaging such as “avoid inactivity,” and “some physical activity is better than none,” yet there is no clear, numerical target for LIPA. Importantly, although there is a lack of trial‐based evidence to underpin numerical targets for LIPA, substitution analyses suggest replacing sedentary time with 30 or more minutes of LIPA per day is associated with health benefits; meta‐analytic evidence from three prospective cohort studies indicated replacing sedentary time with 30 min of LIPA per day lowered the associated risk of cancer mortality by 9%.^56^ In another prospective cohort study, among adults aged 50–79 years, utilizing an isotemporal substitution model,^57^ replacing 30 min of sedentary time per day with LIPA was associated with a 20% reduction in mortality at 5 years; replacing 60 min of sedentary time per day was associated with a 40% reduction.^23^ In cross‐sectional findings, odds of metabolic syndrome were reduced 4% for every 30 min of LIPA per day.^58^ LIPA has also shown initial promising effects on brain health as well. Among older adults aged ≥60 years, engaging in higher levels of LIPA was associated with an approximately 60% reduced odds of decreased cognitive function scores over 8 years,^59^ and among those not meeting guidelines, each additional hour spent in LIPA was associated with approximately 1.4 years less brain aging indicated by MRI markers.^60^ Although LIPA alone may not achieve the same health benefits as meeting MVPA guidelines, it may have many diverse positive health effects among older adult breast cancer survivors with obesity.

### 1.6 Proposed agenda for MVPA and LIPA among older adult breast cancer survivors with obesity

Clearly, there is a need to identify the effects of LIPA on an array of health outcomes across the translational science spectrum. Beyond investigations of the chronic effects of physical activity over weeks and months, laboratory‐based trials of acute responses to single bouts of light/moderate/vigorous‐intensity exercise could elucidate dose–response relationships to physiological and psychological responses. Among the general population, meta‐analytic evidence shows acute bouts of exercise (all intensities) elicit a small‐to‐moderate positive effect on many health outcomes, including post‐bout cognitive function (SMD = 0.11),^61^ state anxiety (SMD = 0.16),^62^ and positive activated affect (SMD = 0.47).^63^ Although understudied compared to MVPA, acute bouts of LIPA showed similar small‐to‐moderate magnitude improvements in post‐bout cognitive function (SMD = 0.17), state anxiety (SMD = 0.08), and positive activated affect (SMD = 0.45).^61^, ^62^, ^63^

#### 1.6.1 Clinical and implementation

Clearly, there is a need to identify the effects of LIPA on an array of health outcomes across the translational science spectrum. RCTs examining the chronic effects of MVPA and LIPA on an array of health outcomes on this population, in pursuit of identification of a minimum effective dose, are of course warranted, and highlighted as an overarching research need by the 2018 US Physical Activity Guidelines Advisory Committee Scientific Report.^8^ Further, it is important to identify efficacious LIPA interventions that are translatable and effective in real‐world settings. Three‐armed RCTs with a MVPA arm, LIPA arm, and control condition could demonstrate the comparative acceptability of LIPA interventions. Further research involving novel approaches to increasing LIPA, including targeting interrupting sedentary time, are also warranted. If pilot LIPA RCTs are shown to be efficacious, pragmatic study designs are needed to identify if LIPA interventions actually increase LIPA, and work in real‐world settings.

### 1.7 Limitations

Based on the disparate nature of research examining older adults, individuals with obesity, cancer survivors, and physical activity, there is limited evidence specifically pertaining to older adult breast cancer survivors. Inferences made from this adjacent literature may not be translatable to older adult breast cancer survivors with obesity. It is also possible that one of these characteristics, that is age, obesity, or breast cancer survivorship, factors more into the practicality and pragmatism of prescribing LIPA or MVPA interventions. Conversely, LIPA could be a practical and pragmatic intervention for obese breast cancer survivors of all ages, or older breast cancer survivors of any BMI.

## 2 CONCLUSION

The growing population of older adult breast cancer survivors with obesity face several poor health outcomes. Guidelines‐based levels of MVPA undoubtedly reduce risk, and improve health, yet a meaningful proportion of survivors may be unlikely to engage in, and especially maintain guidelines‐based levels of MVPA. Therefore, further research of LIPA is warranted, and LIPA prescriptions could be a pragmatic strategy to more effectively promote health‐enhancing physical activity to this complex population.

## AUTHOR CONTRIBUTIONS

All authors were involved in the conceptualization, writing, review, and editing. All authors have given final approval of the version to be published.

## FUNDING INFORMATION

MC is supported by the Four Diamonds Research Funds, Penn State College of Medicine. CKB is partially supported by a National Cancer Institute K07 grant CA215937. JGH was supported by National Institutes of Health/National Institute on Aging R00AG056670. SMB is supported by a Mentored Research Scholar Grant in Applied and Clinical Research, MSRG‐18‐136‐01‐CPPB, from the American Cancer Society (ACS). MP is supported by a National Cancer Institute F32 grant F32CA247263.

## CONFLICT OF INTEREST

DEC serves on a digital clinical trial advisory board for WW, and consults on physical activity research at CJE Senior Life.

## Data Availability

Data sharing is not applicable to this article as no new data were created or analyzed in this study.
